# Eliminating Non-linear Raman Shift Displacement Between Spectrometers via Moving Window Fast Fourier Transform Cross-Correlation

**DOI:** 10.3389/fchem.2018.00515

**Published:** 2018-10-25

**Authors:** Hui Chen, Yan Liu, Feng Lu, Yongbing Cao, Zhi-Min Zhang

**Affiliations:** ^1^School of Pharmacy, Second Military Medical University, Shanghai, China; ^2^Department of Vascular Disease, Shanghai TCM-Integrated Hospital, Shanghai University of Traditional Chinese Medicine, Shanghai, China; ^3^Quality Control Department, Shanghai Diracarta Biomedical Technology Co., Ltd, Shanghai, China; ^4^Department of Foundation and New Drug Research, Shanghai TCM-Integrated Institute of Vascular Disease, Shanghai, China; ^5^College of Chemistry and Chemical Engineering, Central South University, Changsha, China

**Keywords:** Raman instruments, shift correction, cross-correlation, fast fourier transform, moving window

## Abstract

Obtaining consistent spectra by using different spectrometers is of critical importance to the fields that rely heavily on Raman spectroscopy. The quality of both qualitative and quantitative analysis depends on the stability of specific Raman peak shifts across instruments. Non-linear drifts in the Raman shifts can, however, introduce additional complexity in model building, potentially even rendering a model impractical. Fortunately, various types of shift correction methods can be applied in data preprocessing in order to address this problem. In this work, a moving window fast Fourier transform cross-correlation is developed to correct non-linear shifts for synchronization of spectra obtained from different Raman instruments. The performance of this method is demonstrated by using a series of Raman spectra of pharmaceuticals as well as comparing with data obtained by using an existing standard Raman shift scattering procedure. The results show that after the removal of shift displacements, the spectral consistency improves significantly, i.e., the spectral correlation coefficient of the two Raman instruments increased from 0.87 to 0.95. The developed standardization method has, to a certain extent, reduced instrumental systematic errors caused by measurement, while enhancing spectral compatibility and consistency through a simple and flexible moving window procedure.

## Introduction

Over the last few decades, the use of Raman spectroscopy in combination with chemometric methods has increased significantly for analysis of pharmaceutical products (Sacré et al., 2010; Dégardin et al., 2011; Loethen et al., 2015), detection of food adulteration (Zou et al., 2009; Cheng et al., 2010), and other applications (Mrozek et al., 2004; Taleb et al., 2006; Muehlethaler et al., 2011). Raman spectroscopy is a powerful tool for sample analysis and benefits from several advantages such as high speed, simplicity, non-destructive nature, and cost-effectiveness. To date, it has been extensively applied in pharmaceutical analysis by constructing multivariate calibration models. However, these models will be invalid if an existing calibration model is applied to spectra that are collected on a different occasion or a separate instrument, or when the response of an old instrument suffers from variations (Du et al., 2011; Brown, 2013). These variations may, if left untreated, dominate the calibration models, thereby making analysis of samples impractical. Consequently, chemometric techniques have been used to circumvent these problems through instrumental transfer or standardization so as to isolate and compensate for any instrumental and environmental variations.

A number of methods, including both instrumental transfer and standardization, have been discussed in the literature (Wang et al., 1991, 1992; De Noord, 1994; Mann and Vickers, 1999; Nguyen Quang et al., 1999; Hutsebaut et al., 2005; Kompany-Zareh and van den Berg, 2010; Rodriguez et al., 2011b; Weatherall et al., 2013). The direct standardization (DS) and piecewise direct standardization (PDS) developed by Wang et al. (1991, 1992) are the most extensively used procedures for spectral response standardization. Using the PDS method, Gryniewicz-Ruzicka (Gryniewicz-Ruzicka et al., 2011) obtained a very low detection limit for diethylene glycol in pharmaceutical-grade glycerin by using five portable Raman spectrometers. This method, however, requires the user to measure several standards prior to analyzing samples. In addition, both the use of the moving window strategy and the selection of principal components have a noticeable impact on the performance of PDS, which needs to be determined carefully. Furthermore, neither the DS nor the PDS method can deal with different (i.e., non-linear) shifts in the peaks in Raman spectra. It is worth mentioning that in contrast to the various instrumental spectral responses, Raman shift inconsistencies arise mainly from different charge-coupled device (CCD) detectors (Vickers and Mann, 1999). Nonetheless, the use of inconsistent spectra will diminish significantly the predictive power of a calibration model. As a result, the removal of Raman shifts or wavelength inconsistencies for spectra synchronization has become a particularly significant aspect of Raman spectroscopy analysis. In 1996, a mathematical procedure to correct wavelength drifts to synchronize Raman spectra was presented by Booksh et al. (1996). Typically, empirical data are required to select a number of principal components and channels to increase the synchronization precision. Westad and Martens (1999) developed a more general concept of shift determination and tested it on Raman spectra. The results revealed, however, that the spectra were not reproduced exactly after removal of peak drifts exceeding a discrete spectral resolution. Hutsebaut et al. (2005) used a Raman shift standard scattering (SSS for short) method in combination with a linear fitting to determine shift drifts between measured Raman peak and reference positions. A similar approach was used by Rodriguez et al. (2011b) to transfer Raman spectral libraries among instruments. Nevertheless, the use of Raman shift standards is inappropriate for in-line monitoring applications as a result of the difficulties associated with incorporating one or more of the materials proposed as shift standards in a system for in-line measurements. Recently, another approach for the removal of disturbing factors in the CCD responses and instrumental apparatus functions was proposed by Weatherall et al. (2013). Unfortunately, the use of *baselineWavelet* continuous wavelet transform as a function to identify major peaks' positions accurately requires idealized line profiles of the corresponding peaks, which is not practical for real Raman spectra. In addition, several parameters that influence the final results, such as the width of the window and the choice of the signal-to-noise threshold, need to be specified, mostly by the users.

As a result of the multifarious theoretical and practical limitations of the existing instrument standardization methods (Chen et al., 2015), there is a significant demand for methods that are easier to implement (i.e., fewer or even no tunable parameters required) in order to acquire better analytical performance. Accordingly, we introduced a cross-correlation method in order to address the problems (such as tunable parameters, need idealized line profiles, etc.) discussed above. Generally, in signal processing, cross-correlation is a measure of similarity of two waveforms as a function of a time-lag applied to one of them, and is also known as a sliding dot product or sliding inner-product (Welch, 1974; Goshtasby et al., 1984). When coupled with fast Fourier transform (FFT) algorithms, the efficiency of FFT can be exploited in the numerical computation of cross-correlations, accelerating thus the convolution calculation (Bracewell, 1980). FFT cross-correlation may therefore be the fastest method in signal processing for shift correction (Bergland, 1969), and benefits from many advantages such as high speed and accuracy. Moreover, it also eliminates the requirement for alignment parameters. Previously, two alignment methods were proposed to estimate the shifts between segments in large chromatographic and spectral datasets, namely, peak alignment by FFT (Wong et al., 2005b) and recursive alignment by FFT (Wong et al., 2005a). However, these two methods move segments by insertion and deletion of data points at the start and end of segments, without considering peak information, which may cause changes in the shapes of peaks by introducing artifacts and removing peak points. Zhan et al. (Zhang et al., 2012) developed another method, known as the multi-scale peak alignment (MSPA) method, to synchronize peaks against a reference chromatogram (aligning peaks from large to small scales), which is accelerated by the application of FFT cross-correlation while preserving peak shape during synchronization. Similarly, Li et al. (2013a) developed a moving window FFT cross-correlation (MWFFT) method to effectively synchronize high-throughput chromatograms without segment size optimization. However, the Raman spectra profiles were different from the chromatograms, which required peak fitting to obtain perfect profiles and a precise Raman shift.

In the present work, the MWFFT was improved and subsequently applied to spectral standardization to address the issues associated with spectral drifts in Raman spectrometers. The performance of this method was compared to that of the SSS method (Hutsebaut et al., 2005) by using two Raman datasets from primary and secondary spectrometers. The aim of our study was to make the MWFFT as a powerful and practical method for standardization across Raman spectrometers, which can be easily implemented and well-suited for solving Raman shifts displacements between spectrometers.

## Materials and methods

### Standards and samples

Standards (acetaminophen and cyclohexane) were provided by the National Institute for the Control of Pharmaceutical and Biological Products. Pharmaceutical tablets (listed in Table 1) from five different manufacturers were provided by the Shanghai Institute for Food and Drug Control.

**Table 1:** Correlation coefficients of drug tablets before and after shift correction.

**Drugs**	**Batches**	**i-Raman & GemRam**		
		**r^u^**	**R^s^**	**R^m^**
Acyclovir tablets	20100301	0.9424	0.9893	0.9905
	20120102	0.9430	0.9888	0.9906
	130302	0.9186	0.9567	0.9570
	20111201	0.9172	0.9593	0.9605
	20101102	0.9027	0.9585	0.9619
	20110501	0.9358	0.9903	0.9932
	20101103	0.9356	0.9906	0.9938
	20100901	0.9422	0.9923	0.9944
	20110401	0.9377	0.9914	0.9942
	20120101	0.9398	0.9914	0.9937
	100301R	0.9435	0.9924	0.9944
	090601P	0.9365	0.9899	0.9927
	110101	0.9479	0.9932	0.9945
	100101P	0.9477	0.9915	0.9923
	091101P	0.9489	0.9900	0.9904
Captopril tablets	20101009	0.8964	0.9597	0.9644
	090406	0.9178	0.9672	0.9681
	63120501	0.8776	0.9642	0.9692
	110804	0.8919	0.9604	0.9644
	63120401	0.8769	0.9592	0.9620
	110202	0.9131	0.9666	0.9680
	63111001	0.9078	0.9770	0.9796
	110805	0.9196	0.9725	0.9747
	090404	0.9093	0.9658	0.9684
	121003	0.8918	0.9668	0.9720
	63120301	0.8843	0.9621	0.9642
	110901	0.9096	0.9489	0.9509
	090307	0.8923	0.9535	0.9558
	20101006	0.8970	0.9663	0.9714
	63110702	0.8704	0.9490	0.9522
	110801	0.9062	0.9789	0.9804
	20110515	0.8954	0.9669	0.9679
	20101005	0.9060	0.9517	0.9518
	110506	0.9213	0.9546	0.9560
	110702	0.9311	0.9701	0.9717
	110903	0.9271	0.9573	0.9582
	110804	0.9181	0.9543	0.9564
	110604	0.9274	0.9649	0.9651
	110803	0.9084	0.9506	0.9512
	20101004	0.9254	0.9503	0.9503

u Correlation coefficient before shift correction;

s Correlation coefficient by SSS;

m *Correlation coefficient by MWFFT*.

### Raman spectrometers

Two Raman instruments with an excitation wavelength of 785 nm were used, and their physical parameters are listed in Table 2. In this work, the i-Raman is regarded as the “master” (primary) instrument, while the GemRam is regarded as the “slave” (secondary) instrument.

**Table 2:** Physical parameters for the two Raman spectrometers used in this work.

**Spectrometer**	**Manufacturer**	**Laser power (mw)**	**Spectral range (cm^−1^)**	**System resolution (cm^−1^)**	**CCD pixel number**
i-Raman	B&W Tek Inc	100	175–2700	3	2048
GemRam	B&W Tek Inc	100	175–2700	3.5	2048

The integration times of the standards and drugs were of 2 and 3 s, respectively. Unless stated otherwise, six Raman spectra were collected for each drug during the experiment. It is worth noting that the final spectrum of each drug was calculated as the average of spectra collected from a variety of positions. Moreover, only the spectral region containing the most abundant information (i.e., 300–1,700 cm^−1^) was used in subsequent data analysis.

### Cross-correlation

**Figure 1:**
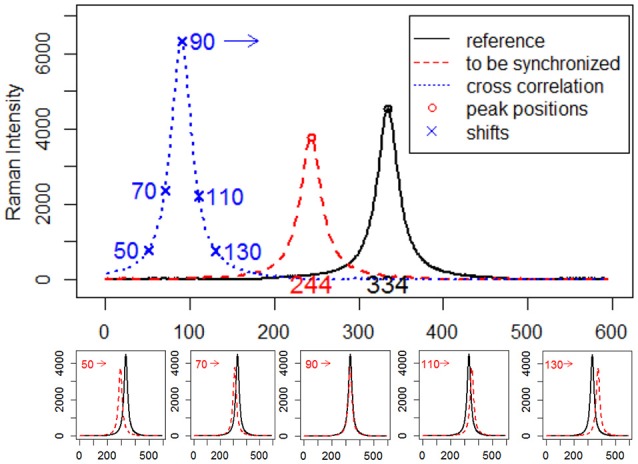
Estimation of displacements between simulated Raman spectra by cross-correlation.

In signal processing, cross-correlation is a standard technique to calculate the similarity between and estimate the linear shift of two signals as a function of one relative to the other, which is also known as the sliding dot product. It is obvious that any changes involving the shifting of one signal will affect the correlation coefficient calculated for any combination of two signals that includes this shifted signal. For two discrete signals such as those in the Raman spectra, the cross-correlation is defined as:

(1) c(j)=∑i(r(i)−r¯)(s(i+j)−s¯)∑i(r(i)−r¯)2∑i(s(i+j)−s¯)2

where r is the reference signal, s is the signal to be synchronized, c is the cross-correlation values for all lags. As a simple example, consider two simulated Raman spectra *r* and *s* that differ only by a known displacement of 90 points along the x-axis. We can determine by how much *s* be shifted along the x-axis in order to maximize its similarity to *r* by using cross-correlation. The above formula slides *s* along the x-axis, calculating the sum of their product at each position. When the value of *c* is maximized, i.e., the signals match well due to peak synchronization, they make the most significant contribution to the sum of their product. A visual description of the calculation procedure of cross-correlation and estimation of shifts between signals via cross-correlation is shown in Figure 1.

### Moving window FFT cross-correlation

The FFT is typically used to calculate the cross-correlation between 1D and 2D signals (Papoulis, 1962; Cooley et al., 1969; Dutt and Rokhlin, 1993). In the present work, FFT was used to increase the speed of cross-correlation between two datasets, in which one signal may be shifted relative to another. In addition, and perhaps more significantly for its application to the spectral synchronization problem, FFT cross-correlation is not heuristic and thus can identify consistently the best match between signals by finding the maximum correlation coefficient (Wong et al., 2005b).

**Figure 2:**
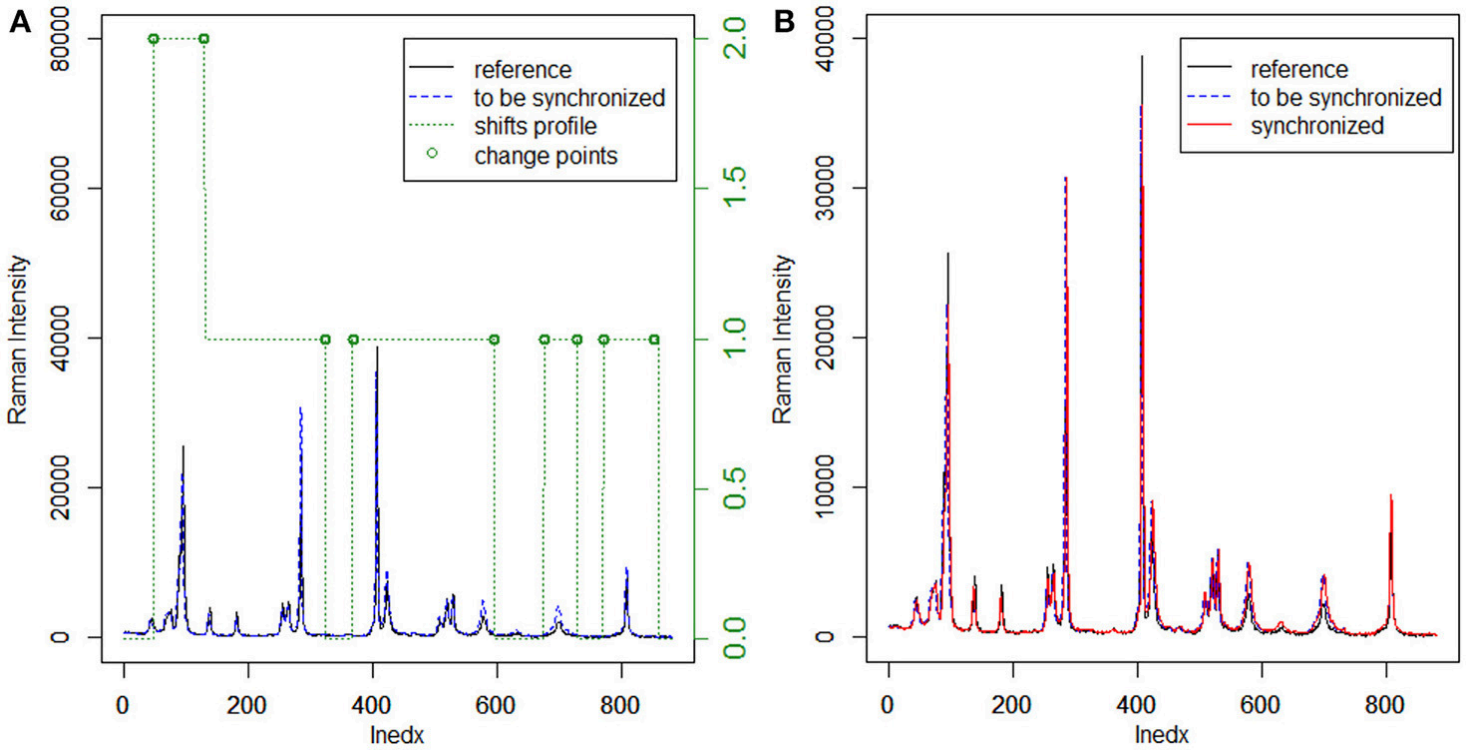
Application of MWFFT to synchronize Raman spectra: **(A)** estimation of nonlinear shift profile between two spectra. **(B)** The continuous regions are moved around change points to obtain the synchronized spectrum.

Usually, the cross-correlation method can only estimate linear shifts between Raman spectra. However, Raman shift displacements are often non-linear in real samples. Consequently, we adopted the moving window procedure in this work to address this problem. In this procedure, the shifts relative to the reference can be estimated by FFT cross-correlation, allowing us to obtain the shift profiles of all samples. Furthermore, MWFFT can be implemented and optimized simply and effectively only if a moving window of appropriate size is utilized. With a window moving from the beginning to the end of the two spectra, one can obtain a matrix of shift points. Accordingly, the shift profile can be obtained by calculating the mode value of each column of the shift matrix. Figure 2A shows an example Raman shift profile estimated by using the moving window strategy and FFT cross-correlation. It is apparent from the obtained shift profile that non-linear shifts exist across the entire spectral region, while the change points are observed in two regions with different shifts. By moving the continuous region around the change points, the synchronization procedure can be finished smoothly to obtain the synchronized spectrum, which can be seen in Figure 2B, with all the non-linear shifts successfully synchronized.

## Results

**Figure 3:**
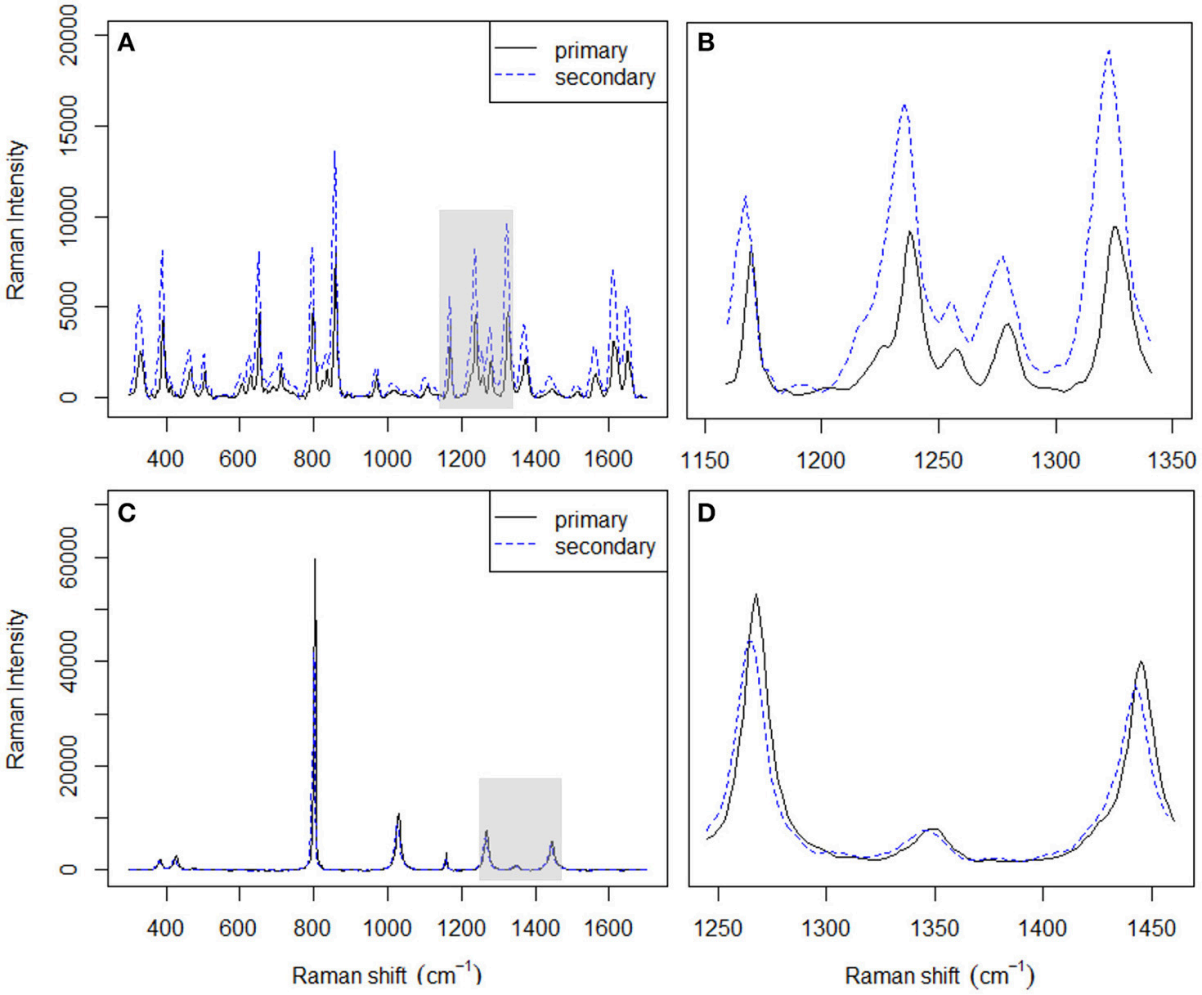
Spectra of acetaminophen **(A)** and cyclohexane **(C)** acquired on two different instruments. Magnified spectral differences in **(B,D)** correspond to the shaded areas in **(A,C)**, respectively.

There are two common ways of correcting the x-axis in Raman spectrometers (McCreery, 2005). The first one is to simply use the SSS method (Hutsebaut et al., 2005); the second one is based on absolute frequency calibration using the emission line spectra of gases. The SSS method, which requires the acquisition of Raman spectra of common materials with well-established Raman shift peak frequencies in order to correct the Raman shift axis directly, is used as a comparative method in this work. Several well-known Raman shift chemical standards, namely, cyclohexane and acetaminophen, are chosen over others for this study since their spectral combination can provide more signals in the region from 300 to 1,700 cm^−1^ (see Table 3). The left panel in Figure 3 shows the spectra acquired for the used chemical standards on two instruments, while the right panel shows a plot of their differences. It should be noted that when the SSS method was used, the spectra acquired on the primary instrument were regarded as the reference, i.e., the peak positions in these spectra were used for synchronization. The relevant peak positions obtained on the secondary instrument are compared to those obtained on the primary instrument and are subsequently subtracted from the primary peak positions to afford the corresponding shift displacements. Linear fitting is then used to describe the shift displacements between the two instruments. Finally, the shift correction is carried out by linear interpolation.

**Table 3:** Raman shifts (cm^−1^) used to calibrate standard samples.

**Standard**	**Raman shift (±standard deviation)^a^**	
**4-ACETAMIDOPHENOL**		
	329.2 ± 0.5	1168.5 ± 0.6
	390.9 ± 0.8	1236.8 ± 0.5
	465.1 ± 0.3	1278.5 ± 0.5
	504.0 ± 0.6	1323.9 ± 0.5
	651.6 ± 0.5	1371.5 ± 0.1
	710.8 ± 0.7	1515.1 ± 0.7
	797.2 ± 0.5	1561.5 ± 0.5
	834.5 ± 0.5	1648.4 ± 0.5
	857.9 ± 0.5	1278.5 ± 0.5
	968.7 ± 0.6	1168.5 ± 0.6
	1105.5 ± 0.3	1236.8 ± 0.5
**CYCLOHEXANE**		
	384.1 ± 0.8	1157.6 ± 0.9
	426.3 ± 0.4	1266.4 ± 0.6
	801.3 ± 0.96	1444.4 ± 0.3
	1028.3 ± 0.5	384.1 ± 0.8

a *Values as reported by ASTM E1840-96*.

### Synchronization of pharmaceutical datasets

**Figure 4:**
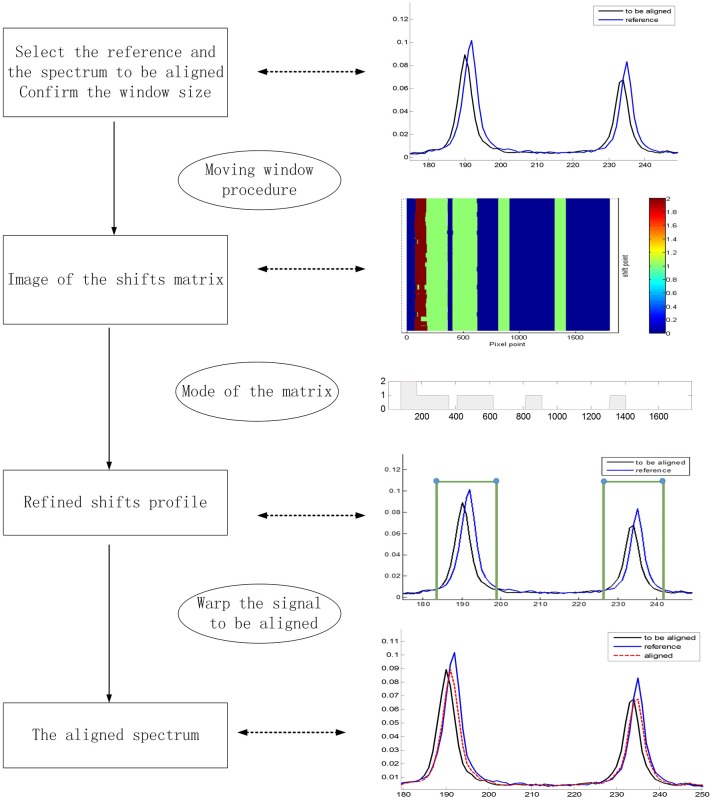
Flowchart describing the framework of MWFFT.

Data synchronization of the raw Raman spectra are presented to evaluate the performance of the MWFFT method (Figure 2). In order to gain further insight into the two shift correction algorithms, and the properties and advantages of MWFFT in particular, different batches of pharmaceutical tablets were examined to verify the practicability and effectiveness of MWFFT. Figure 4 describes the application of MWFFT—each tablet from a total of 40 drugs was analyzed on average six times on two instruments to obtain six different spectra. Subsequently, these spectra were detected for outlier. The average spectrum obtained from six spectra acquired on the primary instrument can be regarded as a reference without outliers. Analogously, we obtained the spectrum of the same tablet on the secondary instrument, and this represents the spectrum to be synchronized. Finally, MWFFT was applied to remove the shift displacements in order to synchronize the spectra across the two instruments.

**Figure 5:**
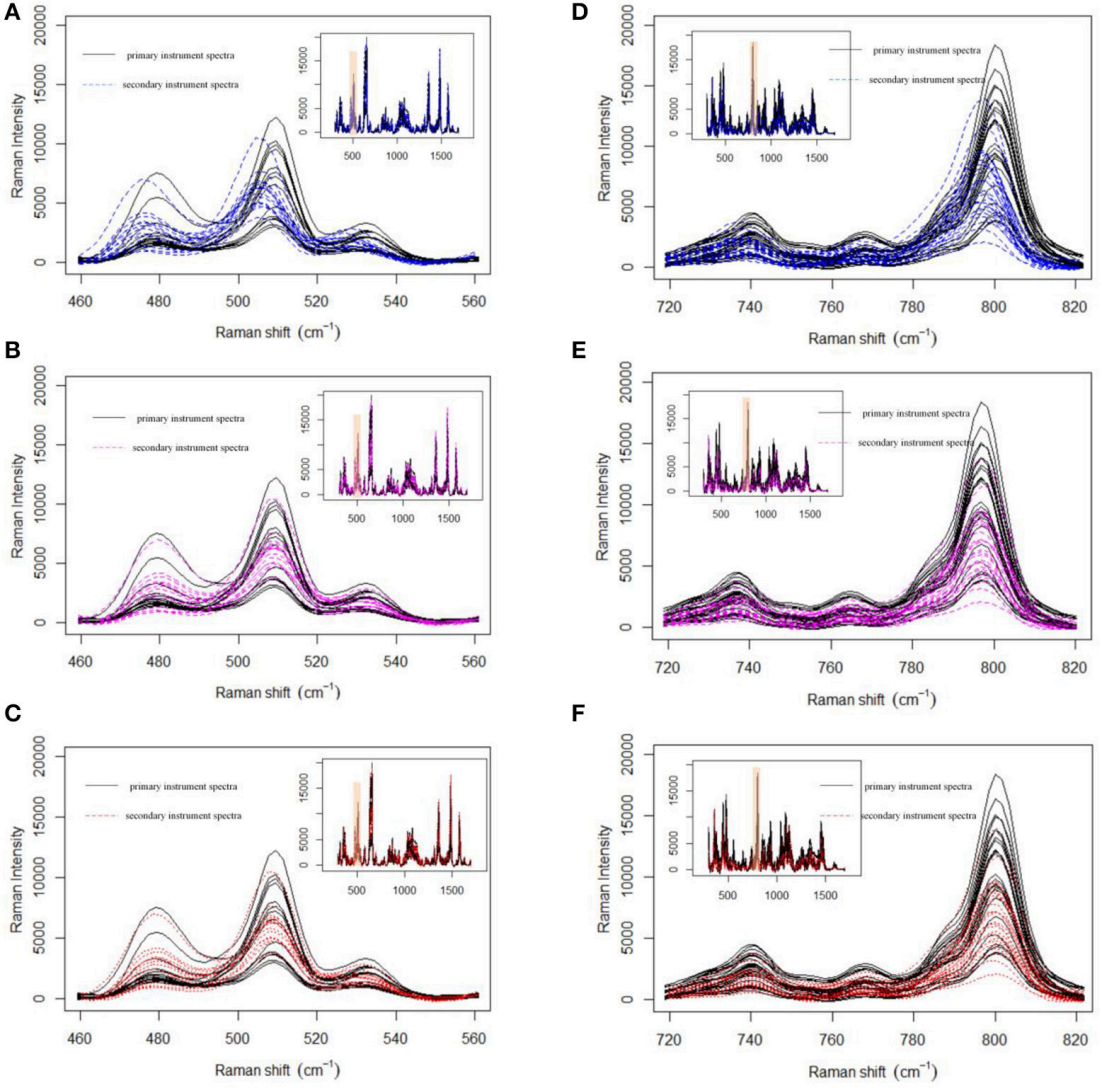
Shift correction data for both acyclovir and captopril datasets with MWFFT and SSS: **(A–C)** acyclovir dataset **(A)** before synchronization, **(B)** synchronized by MWFFT, and **(C)** synchronized by SSS; **(D–F)** captopril dataset **(D)** before synchronization, **(E)** synchronized by MWFFT, and **(F)** synchronized by SSS. The black lines indicate the reference spectra. The inset shows the full Raman spectra, whereas the shaded areas indicate the region magnified in the main panel.

Prior to data analysis, adaptive iteratively reweighted penalized least squares (airPLS) (Zhang et al., 2010a,b; Li et al., 2013b) baseline correction and Savitzky–Golay smoothing (Savitzky and Golay, 1964) (a 9-point wide window and a second-order polynomial) were used in the preprocessing of a variety of pharmaceutical datasets. All processing tasks were implemented on a personal computer (CPU: 2.53G, RAM: 8GB) with MATLAB R2013a. Firstly, we demonstrate the effect of MWFFT by using the pharmaceutical datasets (Figure 5). The primary instrument spectra (black lines) are used as references for synchronization. Figure 5 shows the magnified versions of the sample profiles, focusing on a particular set of peaks in order to allow the performance of the MWFFT method to be evaluated by visual inspection. For the acyclovir and captopril datasets, it can be seen that before synchronization (top panel in Figure 5), the peaks in the spectrum collected on the secondary instrument are de-synchronized with respect to that obtained on the primary instrument, and vary from sample to sample. After synchronization (middle panel in Figure 5) using the MWFFT method, it is apparent that all the spectra are now properly synchronized. This outcome is attributed to the action of the MWFFT method, which appropriately slides the peaks to match the reference spectrum with a window size of 70 points. In addition, for the sake of comparison, the results obtained using the SSS method for the same spectra are displayed in the bottom panel of Figure 5.

### Correlation coefficient after synchronization

The correlation, or distance, between a signal and the reference point is often used as an optimization objective function—when the signals match, the correlation coefficient is maximized. In this case, correlation coefficient can be used to assess the synchronization problem (Lee Rodgers and Nicewander, 1988). Generally, the correlation coefficient is a good descriptor of similarity, with a value of 1.00 indicating a perfect match, while 0 indicates significant dissimilarity. The correlation coefficient is simple to use and possesses several desirable properties, which we discussed in detail in our previous work (Gao et al., 2014). The correlation coefficient can be calculated by using the following equations:

(2) r=∑i=1n(Xip−x¯p)(Xis−x¯s)∑i=1n(Xip−x¯p)2∑i=1n(Xis−x¯s)2

(3) R=∑i=1n(Xip−x¯p)(Xisa−x¯sa)∑i=1n(Xip−x¯p)2∑i=1n(Xisa−x¯sa)2

Here, X^p^ and X^s^ represent the spectra of n drugs measured on the primary and secondary instruments, respectively. Parameters x^p^, ${\bar{\text{x}}}^{s}$, and ${\bar{\text{x}}}^{sa}$ represent the average spectra of X^p^, X^s^, and X^sa^, respectively. X^sa^ indicates the secondary shift corrected spectrum, while r and R denote a similarity between the primary original spectrum and the secondary spectrum (before or after shift correction). The correlation coefficient of each drug's spectrum was calculated, and the results are summarized in Table 1. During the preprocessing, linear interpolation was used to re-compute intensity based on the master Raman shift x-axis in order to unify the spectra obtained using the primary and secondary instruments. It is apparent from Table 1 that the correlation coefficients between the two instruments improved significantly after shift correction.

As can be seen in Table 1, the correlation coefficient assessment prior to the shift correction exhibited a slight variation among different batches of a drug. Nevertheless, these variations are within the three-sigma range. Portable spectrometers are often based on the use of library-based spectral correlation methods (Carron and Cox, 2010), which frequently utilize the hit-quality index (HQI) as the figure of merit to characterize the correlation with each other. The typical minimum threshold that classifies an unknown sample as a “Pass” is 0.95 (Rodriguez et al., 2011a, 2013), which is similar to the correlation coefficient. Clearly, the MWFFT method makes a significant contribution to the level of similarity for the spectra obtained using the slave instrument. The synchronization increased the similarities for all drugs above the verification threshold of 0.95, while the similarity for one captopril tablet remained under 0.95 when the SSS procedure was used. Consequently, it is obvious that the MWFFT method can correct the non-linear shifts successfully, synchronizing thus the secondary spectra to the reference spectra in a time-effective manner. In addition, MWFFT can reduce the systematic differences across spectrometers, which can increase the spectral consistency of different instruments as well as the compatibility with library search. Furthermore, this method can be used as an on-line standardization method across Raman instruments in the future.

## Discussion

### Selection of reference spectrum

**Figure 6:**
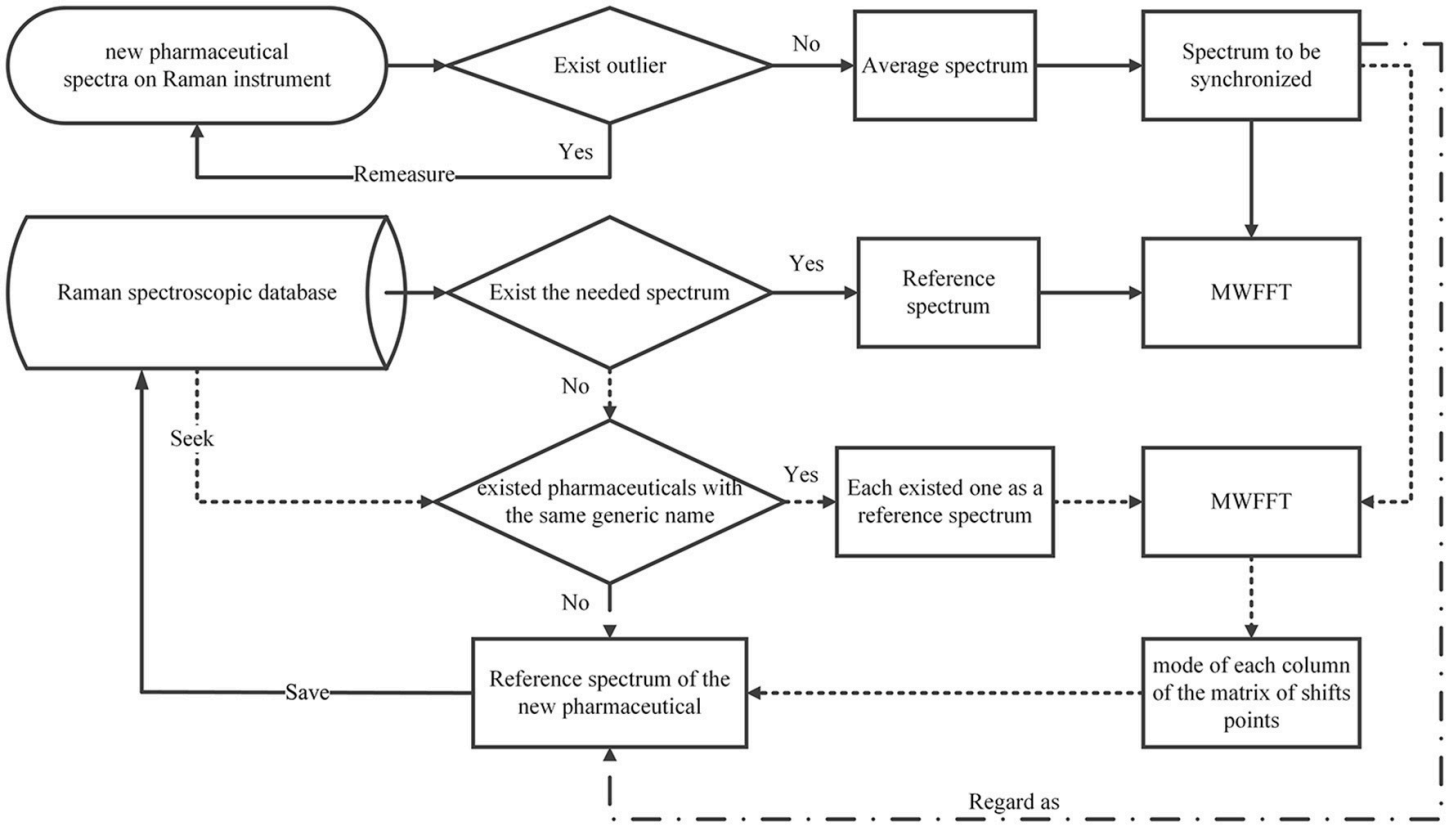
Flowchart of selecting a new drug reference spectrum.

A wide application of the MWFFT method necessitates the selection of an appropriate reference spectrum. When a drug sample is measured on a secondary instrument to obtain an average spectrum for synchronization, its corresponding standard spectrum contained in the existing spectral library can be certainly used as the reference to correct shift displacements. However, when the spectral library does not contain the required reference spectrum, it would be preferable to use the reference spectra of existing drugs with the same generic name in the database in order to obtain a new matrix of shift points. As a result, the shift profile of the new drug can be calculated from the mode of each column of the matrix. Through this profile, one can obtain a new reference spectrum by shift correction, which can be subsequently applied. Otherwise, one can regard the new sample spectrum directly as a reference, and save it in the database for subsequent analysis. The entire procedure is depicted in Figure 6.

### Avoiding peak detection using the moving window strategy

The existing peak detection methods, e.g., the wavelet and ridge line peak picking method, need idealized line profiles of the corresponding peaks in order to detect the displacements accurately, which is not practical for the spectroscopic analysis of real samples. Moreover, several parameters need to be specified with a priori knowledge, which largely influence the final results and can be difficult to implement in C programming language. By contrast, the use of the moving window strategy can allow an estimation of non-linear shifts between spectra flexibly and without peak detection for peak synchronization. With a window that moves from the beginning to the end of two spectra, one can obtain an N-dimensional matrix of shift points, where the data points of a Raman spectrum are N. In this case, the shift profile can be calculated from the mode of each column of the matrix, while the mean and median of the matrix can outline the paths of the shifts. The Raman shift profile of metronidazole tablet is shown in Figure 2A using a green dotted line. It is apparent that the profiles in all regions are corrected by the MWFFT method, meaning this method is sufficiently flexible for estimation of non-linear shifts between spectra.

### Advantages of the MWFFT method

The MWFFT method has several distinctive advantages when compared to the traditional methods as a result of the continuity and redundancy of the moving window procedure. Usually, the direct evaluation of cross-correlation requires O (N^2^) time complexity for a Raman spectrum of length N, which is time-consuming for spectra with thousands of data points. Fortunately, cross-correlation can be calculated by using FFT much more efficiently since it can significantly decrease the time complexity of cross-correlation from O (N^2^) to O (NlogN). The use of the moving window strategy with FFT cross-correlation, with a window size *w*, leads to a time complexity of one window *w*log*w*. Accordingly, the time complexity of MWFFT is N*w*log*w*, where N represents the number of data points in a Raman spectrum.

The MWFFT method evaluates the shift of each point. In the moving window strategy, only one parameter needs to be taken into account, which makes this procedure simple and practical, as there is no need for chemical standards. By contrast, the SSS method requires the use of some chemical standards in order to locate the position of each peak, which is used in turn to obtain the corresponding shift displacement. After the shift of each point is estimated by MWFFT, the points in the spectrum are shifted according to their shifts by insertion and deletion. The present work introduced a change point, i.e., a discontinuity point in the shift profile. It is possible to see that the change points (Figure 2A), around which insertions and deletions occur frequently, are not in the peak region. Consequently, peak distortions can be effectively avoided, allowing the peak shape to be preserved during the synchronization procedure with MWFFT. Overall, the advantages associated with the use of non-linear shift estimation, insertion and deletion around change points, and shape preservation make MWFFT a flexible, rapid, practical, and precise method for correcting shifts in synchronization of Raman datasets.

### Evaluation of the synchronization quality

Generally, Raman spectra will become more consistent, exhibit higher correlation coefficients, and be more similar to each other after a successful synchronization. The correlation coefficient can be used as a criterion for assessing the synchronization quality between the primary and secondary spectra. The synchronized spectra are commonly used to perform library-based searches and are further analyzed by chemometric algorithms. Usually, distance and Euclidean distance in particular (Juday, 1993), can also be a good criterion for evaluating the quality of synchronization. Generally, the more similar the spectra are, the smaller is the Euclidean distance between them, and vice versa. In this work, the mean Euclidean distance (D_*mean*_) is calculated as follows:

(4) $$D_{mean}=1/n\sum_{i=1}^{n}\sqrt{\sum_{j=1}^{k}{\left(X_{i,j}^{p}-X_{i,j}^{s}\right)}^{2}}$$

the rows of matrix X correspond to observations (*n*), while the columns correspond to variables (*k*). ${\text{X}}_{i}^{p}$ and ${\text{X}}_{i}^{s}$ are the *i*th primary (reference) spectrum and secondary spectrum, respectively. It is worth mentioning at this stage that the normalization algorithm (Heraud et al., 2006) is used to scale the spectra within a similar range before calculating the distances. The results are summarized in Table 4. It is apparent that the mean Euclidean distance of the pharmaceutical datasets shift-corrected by SSS and MWFFT were considerably reduced when compared to the uncorrected ones. In addition, for the two datasets, MWFFT performed slightly better than the SSS method in terms of non-linear shift correction.

**Table 4:** Mean Euclidean distances of the used drug datasets shift corrected by SSS and MWFFT.

**Datasets**	**Shift correction methods**		
	**Uncorrected**	**SSS**	**MWFFT**
D*_*mean*_*^ac^	1.9222	1.1637	1.1488
D*_*mean*_*^ca^	2.5565	1.8900	1.8798

ac Mean Euclidean distances of acyclovir datasets;

ca *Mean Euclidean distances of captopril datasets*.

## Conclusions

Methods for the synchronization of spectra are indispensable for successful applications using different spectrometers. In the present work, we used the moving window strategy in combination with FFT cross-correlation to synchronize Raman spectra. This technique, abbreviated as MWFFT, was shown to eliminate accurately and effectively non-linear shift displacements between Raman spectra. Owing to the continuity of the moving window technique, non-linear shifts are corrected and shift profiles are obtained for each spectrum. In general, the use of the FFT cross-correlation methodology is time-saving and results in a significant improvement in speed. Moreover, this method can reduce or even remove systematic differences between Raman spectrometers (a dramatic increase in similarity from 0.87 to 0.95 after synchronization of the spectra between master (primary) and slave (secondary) spectrometers), as well as the compatibility with Raman spectral library. It is better than the SSS method in terms of correcting non-linear shifts and does not require the use of Raman shift standards. These advantages make MWFFT a promising shift correction method that addresses the demand for automated, flexible, rapid, and reliable data preprocessing, which plays an important role in Raman spectroscopy analysis using different spectrometers. Finally, MWFFT can be easily implemented with C and C++ programming languages (available as open source package at http://code.google.com/p/mwfft), which may be well-suited to solving the Raman shift displacements between spectrometers in the fields that rely heavily on the use of Raman spectrometers.

## Ethics statement

The experimental protocol was approved by the Research Ethics Committee of The Second Medical University and Shanghai University of Traditional Chinese Medicine, The findings and conclusions in this article have not been formally disseminated by the State Food and Drug Administration and should not be construed to represent any agency determination or policy.

## Author contributions

HC designed and carried out experiments. Z-MZ and YL assisted with analyzing the results and discussions. HC and Z-MZ wrote the manuscript. FL reviewed and edited the manuscript. YC reviewed and checked our manuscript, gave constructive amendments to the text, and also approved the version to be published.

### Conflict of interest statement

HC is employed by the company Shanghai Diracarta Biomedical Technology Co., Ltd. The remaining authors declare that the research was conducted in the absence of any commercial or financial relationships that could be construed as a potential conflict of interest.
